# Epigenetic reprogramming in pancreatic premalignancy and clinical implications

**DOI:** 10.3389/fonc.2023.1024151

**Published:** 2023-02-16

**Authors:** Wei Zhang, Tingting Jiang, Keping Xie

**Affiliations:** ^1^ Center for Pancreatic Cancer Research, School of Medicine, The South China University of Technology, Guangzhou, China; ^2^ Department of Pathology, School of Medicine, The South China University of Technology, Guangzhou, China

**Keywords:** pancreatic premalignancy, detection, therapy, epigenetics, pancreatic cancer

## Abstract

Pancreatic cancer (PC) is the most lethal human cancer, with less than 10% 5-year survival. Pancreatic premalignancy is a genetic and epigenomic disease and is linked to PC initiation. Pancreatic premalignant lesions include pancreatic intraepithelial neoplasia (PanIN), intraductal papillary mucinous neoplasm (IPMN), and mucinous cystic neoplasm (MCN), with pancreatic acinar-to-ductal metaplasia (ADM) as the major source of pancreatic premalignant lesions. Emerging evidence reveals that an epigenetic dysregulation is an early event in pancreatic tumorigenesis. The molecular mechanisms of epigenetic inheritance include chromatin remodeling; modifications in histone, DNA, and RNA; non-coding RNA expression; and alternative splicing of RNA. Changes in those epigenetic modifications contribute to the most notable alterations in chromatin structure and promoter accessibility, thus leading to the silence of tumor suppressor genes and/or activation of oncogenes. The expression profiles of various epigenetic molecules provide a promising opportunity for biomarker development for early diagnosis of PC and novel targeted treatment strategies. However, how the alterations in epigenetic regulatory machinery regulate epigenetic reprogramming in pancreatic premalignant lesions and the different stages of their initiation needs further investigation. This review will summarize the current knowledge of epigenetic reprogramming in pancreatic premalignant initiation and progression, and its clinical applications as detection and diagnostic biomarkers and therapeutic targets in PC.

## 1 Introduction

Pancreatic cancer (PC) is one of the most lethal diseases, with an incidence nearly equal to the mortality; e.g., there are 495,773 new cases and 466,003 deaths worldwide in 2020 (1). The 5-year survival rate of PC patients is less than 10%, the lowest among common cancers (2). The poor patient survival is mainly due to the fact that PC is asymptomatic in the early stages and difficult for early diagnosis. Although improvements in diagnosis, surgical procedure, and adjuvant therapy have contributed to a sustained decrease in the overall mortality rate, the death rate in PC has increased over the past decades (3). The development of minimally invasive biomarker assays for early diagnosis and effective clinical management of PC is urgently needed to reduce high morbidity and mortality.

The PC develops through the accumulation of a series of genomic and epigenomic alterations and progresses into invasive carcinoma. The process can take up to 15–20 years to develop from the occurrence of the first initiating mutational event. Understanding and discovering early PC and its precursor lesions are fundamentally important to effectively control this deadly disease and improve the outcome of patients. It is generally believed that pancreatic acinar-to-ductal metaplasia (ADM) is the major source of pancreatic premalignant lesions (4). ADM is a reversible process during injury or acute pancreatitis and becomes irreversible with chronic pancreatitis or Kras mutations (5). Pancreatic premalignant lesions include pancreatic intraepithelial neoplasia (PanIN), intraductal papillary mucinous neoplasm (IPMN), and mucinous cystic neoplasm (MCN), with PanIN being the most frequent (4, 6). PC is well investigated at the genetic level, while the molecular basis of its precursor, particularly epigenetics, remains to be explored.

Epigenetics studies the inheritance of phenotypes that occur without a corresponding change in DNA sequence. The molecular mechanisms of epigenetic inheritance include DNA methylation, RNA methylation, non-coding RNA, alternative splicing, histone modifications, chromatin remodeling, and phosphorylation (7–11). Epigenetic changes contribute to the silencing of tumor suppressor genes and the activation of oncogenes. Aberrant epigenetic alterations link to many genes with important roles in PC initiation and progression (12, 13). Epigenetic dysregulation is an early event in pancreatic tumorigenesis. The cancer-associated epigenetic changes occur hours after pancreatic injury at the beginning of ADM and before the appearance of widespread PanIN lesions and involve an “acinar-to-neoplasia” chromatin remodeling that leads to the early malfunction of genes that initiate PC (14). PC initiation results from a complex interaction of genetic and epigenetic damage that triggers changes in cell identity and tissue state and leads to a neoplastic cell fate, i.e., from normal lineage-specifying to cancer-defining (14, 15).

In this review, we first present an overview of the basic molecular mechanisms of epigenetic reprogramming in pancreatic premalignancy and describe the potential use of epigenetic reprogramming for early PC detection and targeted intervention.

## 2 DNA methylation in pancreatic premalignancy

**Figure 1:**
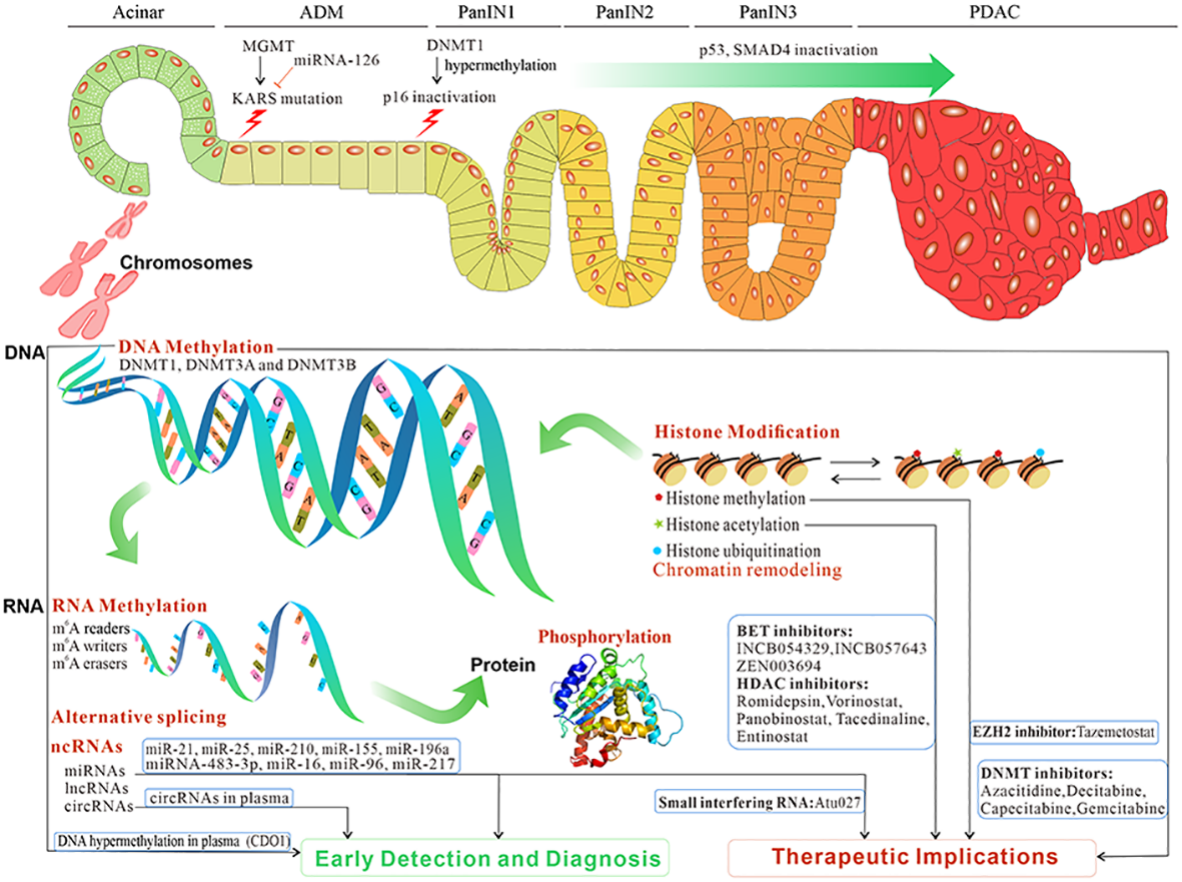
The overview and clinical implications of epigenetic reprogramming in pancreatic premalignancy and PC progression. Epigenetic reprogramming contributes to ADM, and its progression to PanIN and then PC. The initiation and progression of pancreatic premalignancy occur after epigenetic reprogramming such as DNA methylation, RNA methylation, microRNAs, lncRNAs, circRNAs, alternative splicing, histone modification, and phosphorylation. Those epigenetic changes potentially constitute biomarkers for early detection and diagnosis and targeted treatment in PC (data from ClinicalTrials.gov). PC, pancreatic cancer; ADM, acinar-to-ductal metaplasia; PanIN, pancreatic intraepithelial neoplasia.

Epigenetic changes could inhibit tumor suppressor genes and activate oncogenes. DNA methylation is a predominant epigenetic modification and differs in pancreatic neoplasia *vs*. normal pancreas (8, 16, 17). DNA methylation is catalyzed by DNA methyltransferases (DNMTs), which are encoded by a unique family of genes, *DNMT1*, *DNMT2*, *DNMT3*, and *MGMT* (18, 19) (**Table 1**; **Figure 1**).

**Table 1:** Epigenetic alterations in premalignant and malignant pancreatic cancer.

Category	Gene	Molecular function	Molecular phenotype	Sample	Reference
**DNA methylation**	DNMT1	Promoting hypermethylation of tumor suppressor genes and microRNAs	Promoting proliferation, migration, and invasion	Human pancreatic tumors tissue (179 patients)	(20, 21)
	DNMT3A and DNMT3B	Functions as *de novo* methyltransferases	Hypermethylation of promoter CpG-rich regions of tumor suppressor genes	APC^Min/+^Trp53^−/−^ mouse model, PanINs, and PCC tissue	(12, 22, 23)
RNA methylation					
m^6^A writers	METTL3	METTL3 induces m^6^A hypermethylation on the 3′-UTR and enhances its stability	Promote PC progression	Human pancreatic tumors tissue (139 patients)	(13, 24)
	METTL14	METTL14 is the major enzyme that modulates m^6^A methylation	Overexpression significantly increases PC cell proliferation and migration	Human PCC cell lines: PANC-1, MIA PaCa-2, SW1990, AsPC-1, BxPC-3, Capan-2, and Panc 03.27; pancreatic cancers tissue (39 patients)	(25)
	WTAP	m^6^A methylation	Promoting migration and invasion and suppressing chemo-sensitivity to gemcitabine in PC	Human PCC cell lines: MIA PaCa-2, BxPC-3, T3M4, PANC-1, and AsPC-1	(26)
m^6^A erasers	FTO	Removing m^6^A residues in mRNA	FTO is required for PC cell proliferation	Human pancreatic tumors tissue (50 patients)	(27)
	ALKBH5	Globally downregulating RNA m^6^A levels	ALKBH5 overexpression led to a significant reduction in cell migratory and invasion	Human pancreatic tumors tissue (63 patients)	(28)
m^6^A readers	IGF2BP2	IGF2BP2 has the capacity of binding to many transcripts	Promoting aerobic glycolysis and cell proliferation	Human PCC cell lines: BxPC-3, Capan-1, MIA PaCa-2, PANC-1, SW1990; human pancreatic tumors tissue (80 patients)	(29)
MicroRNAs					
**ADM**	hsa-miR-4270 hsa-miR-4462 hsa-miR-3622b-5p hsa-miR-6088 hsa-miR-3934-5p	Target genes: *STAT3*, *PKD1*, RAC1, KRAS, and RHOA	Involved in ADM	Chronic pancreatitis (10 patients)	(30)
	Mir34a	Upregulating the TNFA and IL6	Restraining pre-neoplastic lesions and PCC development	Mir34a^fl/fl^ mouse model	(31)
	let-7b MiR-495	Repressing HNF6 and inhibiting acinar differentiation	Repressing acinar differentiation and inducing hepatic genes	Hnf6^−/−^, Dicer*^loxP/loxP^*, PGK-Cre, Foxa3-Cre, Rosa26R-EYFP, and elastase-Cre mouse model	(32)
	MiR-216a MiR-216b MiR-217	Maybe tumor-suppressive in the pancreas	Acini of the three miRNA KO mice produced greater numbers of ducts, had increased Krt19, and had reduced in genes (amylase 2a and carboxypeptidase A1)	MiR-216a, miR-216b, and miR-217 knockout mouse model	(33)
	MiR-802	Modulating the miR-802-RhoA-F-actin network	Suppressing PC initiation by repressing oncogenic Kras-induced ADM	Mir-802^fl/fl^ mouse model	(34)
**PanIN**	MiR-148a MiR-217 MiR-196 MiR-10b	Oncogenic targets: *ROCK1*, *WNT10 B*, Bcl-2, TGIF2, DNMT3B, and KRAS	Enhancing tumorigenesis	PCC (16 cases), PanIN (5 cases), Chronic pancreatitis (4 patients)	(35)
	MiRNA-148a	Targeting the mRNA of the DNMT3B	Significant downregulation in the PanIN-1B, PanIN-2, and PanIN-3 lesions	PanIN-1A (n = 8), PanIN-1B (n = 11), PanIN-2 (n = 10), and PanIN-3 (n = 10)	(36)
	MiR-483-3p	Expression negatively correlating with Smad4	Overexpressed in PanIN and PCC, promoting PC cell migration and invasion	Human pancreatic tumors tissue (107 patients)	(37)
**IPMN**	MiRNA-101	The loss of miRNA-101 could be a trigger for the EZH2	Tumorigenesis of IPMN	70 IPMN lesions (51 benign and 19 malignant)	(38)
	MiRNA-126	Directly targeting KRAS transcript through its binding site within 3′-UTR	Downregulating miRNA-126 in PCC compared to low malignant	serous microcystic adenomas (SMCA) (n = 7), MCN (n = 6), IPMN (n = 7), Carcinoma-Ex-IPMN (CEI) (n = 9), and PCC (n = 14)	(39)
	MiRNA-155	Acetylation and methylation are upregulated in intraductal papillary mucinous neoplasms (IPMNs)	IPMN malignant transformation	30 IPMN tissue samples	(40)
**MCN**	MiR-224-5p	Directly targeting PTEN	Promoting the proliferation, migration, and invasion	Human pancreatic MCC tumors tissue (4 patients)	(41)
**LncRNAs**	HAND2-AS1	Regulating cytokine–cytokine receptor interaction, PI3K–Akt signaling pathway, calcium signaling pathway, and actin cytoskeleton	Promoting tumorigenesis of IPMN	Human pancreatic tumors tissue (44 patients)	(42)
	LINC00857	Competing endogenous RNA for sponging miR-150-5p	Promoting proliferation and inhibiting apoptosis in PC cells	Human pancreatic tumors tissue (12 patients)	(43)
	DUXAP8	Directly targeting miR-448, and miR-448 directly binds to WTAP	Modulating migration, invasion, and proliferation of PC cells	Human pancreatic tumors tissue (24 patients)	(44)
	MALAT1	Regulating Hippo-YAP signaling and interacting with RNA binding protein HuR	Influencing proliferation, migration, and invasion in PC	Human pancreatic tumor tissues (15 patients)	(45, 46)
	CERS6-AS1	Competitively binding to miR-15a-5p and working as a molecular sponge	Promoting proliferation, migration, and invasion in PC cells	Human PCC tissues (57 patients)	(47)
	TP73-AS1	Binding to miR-128-3p causes GOLM1 upregulation, positively regulating BDH2 expression by sponging miR-141	Promoting PC progression, migration, and invasion	Human pancreatic tumor tissues (116 patients)	(48, 49)
	PART1	Serving as a molecular sponge of miR-122	Reducing cell proliferation and invasion	Human pancreatic tumor tissues (45 patients)	(50)
	LINC00460	MiR-320b directly targets LINC00460	Inhibiting the proliferation, migration, and invasion	Pancreatic cancer cell lines (SW1990, HPAC, PaCa-2, CFPAC-1, and CAPAN-1)	(51)
**CircRNAs**	CircEYA3	As an endogenous miR-1294 sponge to upregulate c-Myc expression	Promoting PC progression	104 PCC tissues	(52)
	CircNEIL3	Regulating the expression of ADAR1 by sponging miR-432-5p to induce RNA editing	Promoting PC progression	Human pancreatic tumor tissues (10 patients)	(30)
	Circ_0075829	Regulating LAMTOR3/p-ERK signaling pathway *via* sponging miR-1287-5p	Suppressing the proliferation, migration, and invasion of PC cells	Human pancreatic tumor tissues (38 patients), cell lines: AsPC, PANC1, MiaPaca-2, SW1990, and BxPC-3	(53)
	CircCCT3	As a sponge for miR-613 facilitating VEGFA and VEGFR2 expression	Promoting cell proliferation, migration, and invasion in PC	Human pancreatic tumor tissues (30 patients), cell lines (Patu8988, SW1990, BxPC-3, and Panc02)	(54)
**Alternative splicing**	hMENAΔv6	hMENAΔv6 upregulation is crucial for SMAD2-mediated TGF-β1 signaling	Promoting TGF-β1-induced EMT	285 primary PCC tissues	(55)
	masTF	masTF lacks exon 5 and has a distinct 93-amino-acid C-terminus	Likely contributing to pancreatic tumor growth	K-ras and Ldlr^–/–^ mouse models	(56)
	FGFR-2 IIIb/IIIc	The alternative splicing of FGFR-2	Promoting proliferation, migration, and invasion	Human pancreatic tumor tissues (117 patients); KLM-1, PANC-1, MIAPaCa-2, PK-1, and PK-8 PCC cell lines	(57, 58)
Histone modification					
**Histone methylation**	NSD1 and SETD2	*NSD1* and *SETD2* genes encoding two histone H3K36 methyltransferases	Playing an important role in PC tumorigenesis and progression	Human pancreatic tissues (190 SETD2 and 192 NSD1)	(59)
	Men1	Mediating the methylation of histone 3 on lysine 4	Accelerating KRAS-induced tumor formation	Men1 conditional knockout mouse model	(60, 61)
	NFATc2	Binding to p15INK4b promoter site and inducing the H3K9 trimethylation	Promoting PC cell growth	Human pancreatic cell lines: IMIM-PC1 and PaTu8988t	(62)
Histone acetylation					
	ACLY	Generating acetyl-CoA and regulating global histone acetylation	Facilitating cell plasticity and proliferation	Acly^PANC−/−^ mouse model	(63)
	HDAC1	Histone deacetylase	Promoting the malignancy of PC	48 PanIN-1A, 103 PanIN-1B, 99 PanIN-2, 30 PanIN-3, 18 IPMA, 10 IPMB, 20 IPMC, and 54 PCC	(64)
Histone ubiquitination					
	Ring1b	Catalyzing histone modification H2aK119ub	Ring1b knockout inhibiting the formation of ADM induced by KRAS	Ring1b^flox/flox^ mouse model	(65, 66)
	BMI1	Catalyzing histone modification H2AK119Ub	Onset of pancreatic carcinogenesis and metastasis	*Bmi1^fl/fl^* mouse model	(66, 67)
Chromatin Accessibility					
	BRG1	SWI/SNF chromatin remodeling complex	BRG1 deletion leading to the formation of IPMN	*Brg1^fl^* mouse model	(68, 69)
	ARID1A	SWI/SNF chromatin remodeling complex	ARID1A deletion accelerating the onset of invasive cancer	Arid1a^flox/flox^ mouse model	(70, 71)
**Phosphorylation**	S6	Ribosomal protein (S6) phosphorylation	Increasing malignant cells of IPMN	39 patients with IPMN, human pancreatic cell lines: BxPC-3, PK-45H, and PANC-1	(9)
	Ezrin	Activated by threonine and tyrosine phosphorylation	The p-ezrin (tyr354) expression in IPMNs is associated with PC invasion	131 IPMNs, 48 PanINs, and 59 invasive ductal carcinomas	(72, 73)

PanIN, pancreatic intraepithelial neoplasia; PCC, pancreatic ductal adenocarcinoma cell; PC, pancreatic cancer; ADM, acinar-to-ductal metaplasia; MCN, mucinous cystic neoplasm; EMT, epithelial–mesenchymal transition.

### 2.1 Expression of DNA methyltransferases

*DNMT1* is the most abundant and primarily a DNA maintenance methyltransferase and is responsible for copying methylation patterns after DNA replication (20). *DNMT1*, *DNMT3A*, and *DNMT3B* mRNA levels significantly increase from normal ducts to PanINs to PC (74). Similar to the mRNA results, DNMT1 protein expression increases from normal ducts with or without inflammation to pre-cancerous lesions (PanINs or IPMNs) to PC (64).

DNMT1 protein overexpression is involved in multistage tumorigenesis of the pancreas. PC patients with high levels of DNMT1 protein expression have a poorer outcome than those with low levels of DNMT1 expression (64, 75). Higher DNMT1 expression correlates with advanced stages of the disease (64). DNMT1 is oncogenic in promoting malignant phenotype and stemness in PC. These effects are achieved *via* promoter hypermethylation of tumor suppressor genes, e.g., cyclin-dependent kinase inhibitors, tumor suppressor microRNAs, and suppressors of epithelial–mesenchymal transition (EMT) (21).

DNMT2 methylates specifically cytosine 38 in the anticodon loop. DNMT2 is also involved in DNA recombination, DNA damage recognition, and mutation repair. The levels of DNMT3A, DNMT3B, and DNMT3L are usually elevated in cancer tissues and cells, in part due to the hypermethylation of promoter CpG-rich regions of tumor suppressor genes (22). DNMT3A and DNMT3B function as *de novo* methyltransferases (20). Patients having PC with higher levels of DNMT3A and/or DNMT3B expression have a poorer prognosis than those with a lower-level expression (74). In addition, the expressions of DNMT1 and DNMT3a in PC tissues are all higher than those in adjacent normal tissues (23, 76). However, the potential changes in their expression and function and underlying mechanisms in pancreatic premalignancy remain to be defined.

*O* ^6^-Methylguanine DNA methyltransferase (MGMT) is another important DNMT and DNA-repair protein that removes mutagen from the *O* ^6^ position of guanine. *O* ^6^-Methylguanine often mispairs with thymine during replication and results in G-to-A point mutations if the adduct is not removed (18). MGMT methylation is associated with mutations of *KRAS*, *TP53*, and *SMAD4* in PC (77). There is approximately 25% loss of MGMT in PC (78) and pancreatic endocrine tumors (79).

### 2.2 DNA methylation of tumor genes

DNA methylation of CpG islands in the promoter regions of tumor-related genes is associated with the silencing of those genes (12). Aberrant DNA methylation is associated with chromosomal instability, while deamination of 5-methylcytosine to thymine increases gene mutagenicity (80). Aberrant methylation of the *ppENK* gene increases progressively from PanIN-1A and PanIN-3 to PC. Aberrant methylation of *p16* gene is also increased from PanIN-1A to PanIN-3. The methylation of *p16* and *ppENK* genes increases with PanIN grades, suggesting that methylation of these genes is an intermediate or late event during pancreatic carcinogenesis (8). Moreover, the majority of IPMNs exhibit hypermethylation of CpG islands. Hypermethylation of *p16* and *ppENK* occurs significantly higher in high-grade IPMNs than in low-grade IPMNs. The methylation of multiple CpG islands is one of the important pathways linked to the development and progression of IPMNs (16).

The aberrant methylation is identified in the majority of the earliest PanIN-1A lesions. *NPTX2* increases in methylation from PanIN-1 to PanIN-2, while *SARP2*, *Reprimo*, and *LHX1* increase in methylation from PanIN-2 to PanIN-3. The aberrant hypermethylation of CpG islands occurs in the early stages of PanINs, and its prevalence progressively increases during tumor progression (17). WNK2 is a cytoplasmic serine–threonine kinase. WNK2 interferes with the activation of the MEK1/ERK1/2 MAP kinase pathway and inhibits cell proliferation. WNK2 hypermethylation is higher in PanIN lesions than in surrounding tissues (81).

## 3 RNA methylation in pancreatic premalignancy

**Figure 2:**
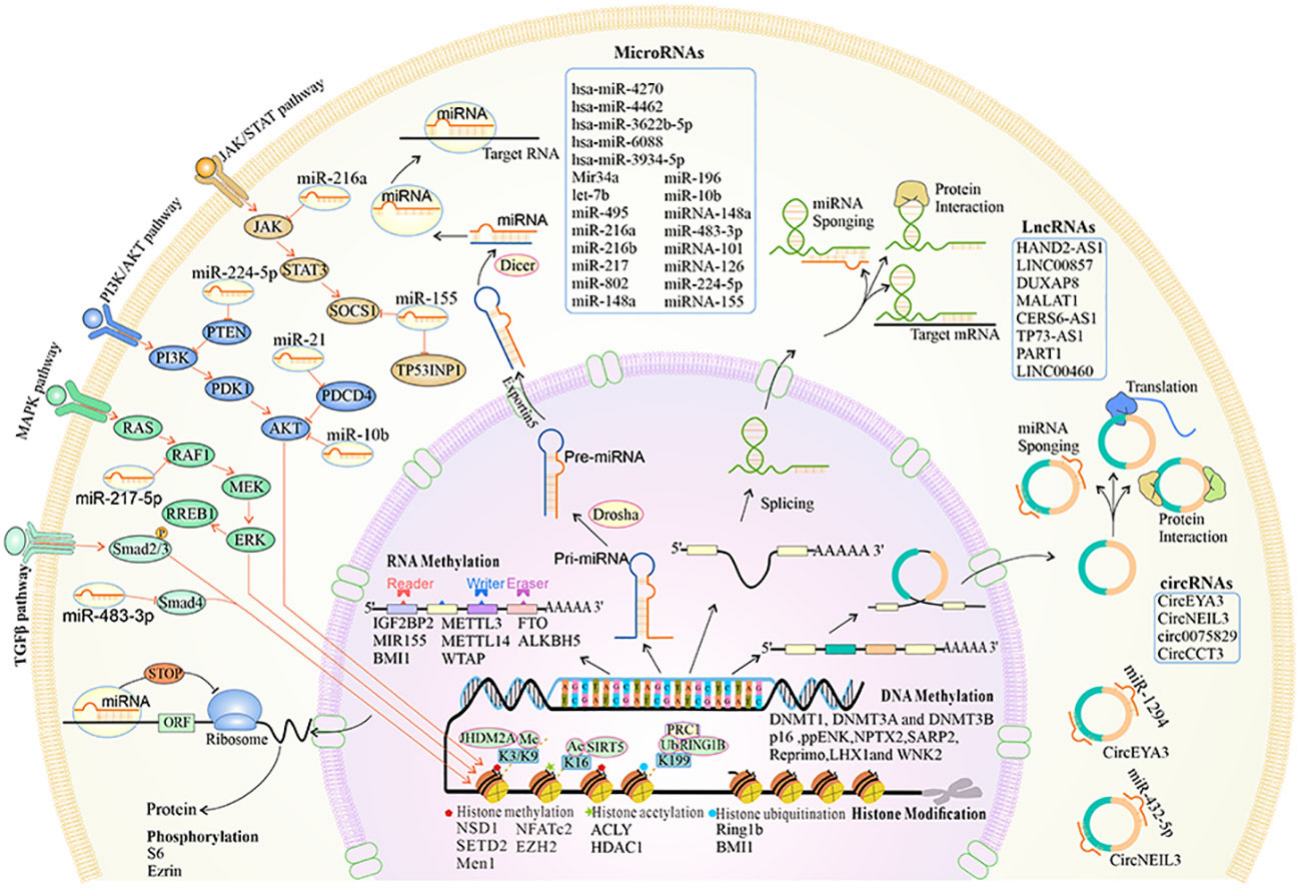
The overview of the biogenesis, functions, and mechanisms of epigenetic reprogramming in pancreatic premalignancy. At the chromatin level, histone methylation, acetylation and deacetylation, and histone ubiquitination occur. DNA and RNA can be modified by methylation. Alternative splicing leads to different isoforms of mRNA and proteins. MiRNAs can regulate different key downstream gene targets that mediate cellular signaling pathways. The lncRNAs are cell type-specific and cell state-specific. The lncRNAs can act as miRNA sponges, regulate mRNA stability, and interact with proteins. The circRNAs are transcribed by the RNA polymerase and can be processed through alternative splicing of the pre-mRNA. The circRNAs can act as miRNA sponges, interact with proteins, and translate into proteins.

*N* ^6^-Methyladenosine (m^6^A) is the most common modification that exists in mRNAs and long non-coding RNA (lncRNAs) (11). The m^6^A modification system is comprised of m^6^A writers (WTAP, METTL3, and METTL14), m^6^A erasers (ALKBH5 and FTO), and m^6^A readers (YTH domain-containing family proteins, IGF2BP1, IGF2BP2, and HNRNPA2B1) (11, 82–84). m^6^A has critical roles in tumorigenesis, and alterations in the m^6^A modification system may provide new therapeutic strategies (**Table 1**; **Figures 1**, **2**).

### 3.1 m^6^A writers

METTL3 mRNA and protein are significantly higher in PC than in adjacent tissues. The increased expression of METTL3 is associated with advanced stages and decreased survival (85). Moreover, the m^6^A RNA methylation of *IGF2BP2*, *IGF2BP3*, *METTL3*, *FTO*, *YTHDC1*, and *YTHDF1* is increased, while that of *ALKBH5 METTL16*, *RBM15*, and *ZC3H13* is downregulated (86). Overexpression of METTL3 causes m^6^A modification and an excessive maturation of miR-25-3p. The upregulation of miR-25-3p inhibits *PHLPP2* and activates AKT-p70S6K oncogenic signaling, forming a malignant METTL3–miR-25-3p–PHLPP2–AKT axis and promoting PC development and progression (13).

Leukemia inhibitory factor receptor antisense RNA 1 (*LIFR-AS1*) is a novel cancer-related lncRNA. METTL3 induces m^6^A hypermethylation on the 3′-UTR of *LIFR-AS1* and enhances its stability. *LIFR-AS1* interacts directly with miR-150-5p and indirectly increases VEGFA expression, promoting pancreatic tumorigenesis (24). The nucleobindin 1 (NUCB1), a calcium-binding protein, controls the unfolded protein response. METTL3 mediates m^6^A modification on NUCB1 5′-UTR *via* YTHDF2 and downregulates NUCB1, thus promoting PC cell proliferation (87).

METTL14 is an enzyme that modulates m^6^A methylation. METTL14 overexpression significantly promotes pancreatic tumorigenesis by directly targeting the mRNA of *p53* effector related to PMP-22 (25), MPKα, ERK1/2, and mTOR signaling pathways (88), cytidine deaminase, an enzyme inactivating gemcitabine (89), and Cdc2-like kinases 1/SRSF5/cyclin L2 pathways (24). WTAP plays an important role in transcriptional and post-transcriptional regulation. WTAP is an independent prognostic indicator for PC. The WTAP promotes malignancy and suppresses gemcitabine chemo-sensitivity in PC (26). The pseudogene *WTAPP1* RNA significantly increases in PC and is associated with poor prognosis in patients. Decreasing *WTAPP1* RNA significantly suppresses the growth and metastasis of PC cells (90). However, their involvement in pancreatic premalignancy is unclear.

### 3.2 m^6^A erasers

Fat mass and obesity-associated (FTO) protein is a critical demethylase, or m^6^A eraser, in regulating mRNA stability by removing m^6^A residues in mRNA. The role of FTO in cancer cell homeostasis remains unclear. FTO is required for PC cell proliferation, and its knockdown causes a compromised DNA synthesis and PC cell proliferation but an increase in apoptosis. FTO interacts with MYC and bHLH transcription factors and enhances their stability by decreasing m^6^A levels. Reduced FTO expression results in elevated levels of m^6^A RNA modification in PC. Moreover, FTO demethylates the m^6^A modification of Praja ring finger ubiquitin ligase 2 (*PJA2*), reduces its mRNA degradation, suppresses Wnt signaling, and ultimately inhibits the malignant phenotype of PC cells (27). This novel mechanism for the regulation of PC malignancy by FTO could be a therapeutic target of PC (91).

Alkylation repair homolog protein 5 (ALKBH5) is an m^6^A demethylase. Its overexpression globally downregulates RNA m^6^A levels. ALKBH5 is generally downregulated in PC cells (92). P53-mediated activation of ALKBH5 transcription regulates the m^6^A modifications in a feedback loop (93).

ALKBH5 substrates include those mRNAs encoding mitochondrial iron importers SLC25A37 and SLC25A28 and ubiquitin ligase FBXL5. Downregulation of FBXL5 in tumor samples correlates with shorter patient survival. ALKBH5 overexpression incurs FBXL5-mediated degradation of and a significant reduction in iron regulatory protein IRP2 and the modulator of EMT, SNAI1. ALKBH5 overexpression leads to a significant reduction in intracellular iron levels as well as cell migration and invasion, which could be rescued by FBXL5 knockdown (28). Wnt inhibitory factor 1 (*WIF-1*) is also ALKBH5 target gene. Increased ALKBH5 decreases *WIF-1* RNA methylation and transactivates *WIF-1*, thus inhibiting Wnt signaling and PC (94). ALKBH5 activates *PER1* by m^6^A demethylation in an m^6^A-YTHDF2-dependent manner. Upregulated PER1 reactivates ATM-CHK2-P53/CDC25C signaling and inhibits PC cell growth (93).

ALKBH5 can also demethylate lncRNA *KCNK15-AS1*. Both ALKBH5 and *KCNK15-AS1* are lower in PC tissues than in adjacent normal tissues. *KCNK15-AS1* inhibits the malignancy of PC cells, while ALKBH5 downregulation and then *KCNK15-AS1* downregulation promote PC cells (92). *KCNK15-AS1* overexpression suppresses cell proliferation and EMT while promoting cell apoptosis in PC (92). ALKBH5 induces m^6^A demethylation of *KCNK15-AS1* mRNA and then *KCNK15-AS1* upregulation. Interestingly, *KCNK15-AS1* recruits MDM2 proto-oncogene (MDM2) to promote the ubiquitination of RE1 silencing transcription factor (REST), thus transcriptionally upregulating phosphatase and tensin homolog (PTEN) and attenuating the AKT pathway (95).

### 3.3 m^6^A readers

The m^6^A modifications are recognized by m^6^A-binding proteins, such as YTHDF1/2/3, IGF2BP1/2, and HNRNPA2/B1. These proteins are regarded as “m^6^A readers” (84). Abnormal modifications in the m^6^A levels are closely related to tumorigenesis. Specifically, IGF2BP2 is a member of the IGF2BP protein family. IGF2BP2 has the capacity of binding to many transcripts. The expression of IGF2BP2 is upregulation with a poor prognosis in PC. IGF2BP2 protein is gradually upregulated from normal pancreas, PanIN to PC in the *Kras^G12D^* mouse model. IGF2BP2 promotes aerobic glycolysis and cellular proliferation *via* directly binding to and stabilizing *Glut1* mRNA (29, 96). The hnRNP A2/B1 is upregulated in the PanIN and pancreatic cancer tissues (97). Moreover, hnRNP A2/B1 increases in PC tissues and cell lines (98).

## 4 Non-coding RNA in pancreatic premalignancy

Non-coding RNAs (ncRNAs) include circular RNAs (circRNAs), lncRNAs, and microRNAs (miRNAs) (**Table 1**; **Figure 2**).

### 4.1 MicroRNAs

MicroRNAs are small non-coding RNAs of 20–22 nucleotides. Growing evidence suggests that miRNAs interfere with cellular functions in pancreatic premalignancy and malignancy (41). The direct targeting of miRNAs provides a novel approach to the development of targeted intervention of early PC.

#### 4.1.1 MicroRNAs in ADM

ADM in an inflammatory environment is a precursor of PC. From the miRNA–mRNA networks in the serum, hsa-miR-24-3p, hsa-miR-149-3p, hsa-miR-6785-5p, hsa-miR-4728-5p, hsa-miR-6808-5p, hsa-miR-6779-5p, hsa-miR-6799-5p, hsa-miR-6086, hsa-miR-4722-5p, and hsa-miR-4433a-3p are significantly upregulated, while hsa-miR-5100 is downregulated. hsa-miR-4270, hsa-miR-4462, hsa-miR-3622b-5p, hsa-miR-6088, and hsa-miR-3934-5p are also involved in ADM (30). In *Ptf1a^+/Cre^* and *Kras^+/LSL-G12D^* mice, pancreas-specific loss of Mir34a leads to an accelerated formation of pre-neoplastic lesions and a faster PC development (31).

As a critical component of the miRNA processing machinery, Dicer is an enzyme containing RNase III. Dicer essentially maintains acinar cell identity. Acinar cells without Dicer exhibit increased plasticity, as evidenced by loss of polarity and initiation of ADM and EMT. Moreover, homozygous Dicer deletion accelerates the formation of ADM but not PanIN, whereas heterozygous Dicer deletion accelerates PanIN initiation, suggesting complex roles for Dicer in the regulation of normal and neoplastic pancreatic epithelial identity (99). Let-7b and miR-495 repress HNF6 and express in developing acini. Let-7b and miR-495 expressions are downregulated in Dicer-deficient acini and pancreatitis-induced ADM. Moreover, suppressing let-7b and miR-495 causes similar effects in Dicer-deficient acini and metaplastic cells, i.e., induction of HNF6 and other hepatic genes and suppression of acinar differentiation. This gene network of Let-7b, miR-495, and their targets establish and maintain pancreatic acinar cell differentiation (32).

MiR217HG encodes miR-216a, miR-216b, and miR-217, which are enriched in pancreatic acinar cells. In germline knockout mice of miR-216a, miR-216b, or miR-217, the acini from each of the three miRNA-knockout mice produce many more ducts than those from control mice. There is an increased expression in ductal genes and reduced expression in acinar cells after acinar transdifferentiation. In caerulein-induced acute pancreatitis, miR-216a- and miR-216b-KO mice have more pancreatic duct glands and delayed recovery (33).

MiR-802 is highly expressed in pancreatic acinar cells and silenced during the early stages of pancreatic injury or cellular transformation. Knockout of miR-802 promotes ADM formation in *Ptf1a^+/Cre^;Kras^+/LSL-G12D^* mice. Downregulation of miR-802 activates *Sox9* and enhances the expression levels of ductal identity genes but attenuates the expression of acinar identity genes. MiR-802 also inhibits PC development by suppressing oncogenic *Kras*-induced ADM (34).

#### 4.1.2 MicroRNAs in PanIN

MicroRNA deregulation is a very early event in the progression of PC. MiRNA-21, miRNA-221, miRNA-222, and let-7a are upregulated in PanIN lesions, with peak expression occurring in PanIN-2 and PanIN-3 (100, 101). MiR-148a and miR-217 expression levels are downregulated in PanIN-2 and PanIN-3 and PC, whereas the level of miR-196 is significantly elevated in PC and PanIN-2 and PanIN-3 as compared with normal pancreatic parenchyma. Moreover, miR-10b is highly expressed in PanIN-2 and PanIN-3. These markers could be used as biomarkers to distinguish PC and its precursors from benign lesions (35). MiRNA-196 is most specifically overexpressed miRNA in PanIN-3, which has potential use as a marker for PanIN-3 lesions (102).

The expression of miRNA-148a is significantly downregulated in PanIN-1B, PanIN-2, and PanIN-3 lesions as compared with normal duct and PanIN-1A. The methylation level in the coding region of miRNA-148a is significantly higher in PC samples than in chronic pancreatitis samples. MiRNA-148a downregulation occurs at an early stage of PC initiation and progression (103). MiRNA-148a targets the mRNA of *DNMT3B*. The downregulation of miRNA-148a occurs early in premalignancy lesions, leading to *DNMT3B* upregulation and the inactivation of tumor suppressor genes (36). MiR-155 is significantly overexpressed in both PanIN-2 and PanIN-3, while miR-21 is highly expressed only in PanIN-3 (104). MiR-196b only is expressed in PanIN-3 tissue (102).

MiR-483-3p is overexpressed in PanIN and PC. Moreover, miR-483-3p expression levels correlate with increases in the PanIN grade. Circulating miR-483-3p levels are significantly increased in the serum and serum exosomes of PC patients. Interestingly, serum levels of miR-483-3p can distinguish patients with early-stage PC (≤2 cm) from healthy controls. MiR-483-3p expression negatively correlates with Smad4 expression in PC and PanIN tissues (37).

#### 4.1.3 MicroRNAs in IPMN

The expression of miRNA-101 is significantly lower and *EZH2* is dramatically higher in malignant IPMN than in benign IPMN. The loss of miRNA-101 could be a trigger for the tumorigenesis of IPMN by upregulation of *EZH2* (38). Downregulation of miRNA-126 is detected in PC as compared to low malignant pancreatic benign cystic tumors. MiRNA-126 directly targets KRAS transcript through its binding site within 3′-UTR (39).

In high-risk IPMNs, oncogenic targets are upregulated, e.g., miR-126 (*IRS-1*), miR-130a (*ATG2B* and *MEOX2*), and miR-342-3p (*DNMT1*) (105). MiR-21 and miR-155 are higher in invasive IPMNs than in non-invasive IPMNs and normal tissues. Conversely, miR-101 is lower in invasive IPMNs than in non-invasive IPMNs and normal tissues. High levels of miR-21 are associated with worse overall survival. Patients with high-miR-21 expression also have a shorter median disease-free survival (106). MiR-10a-5p and miR-221-3p are upregulated while miR-148a-3p is downregulated in invasive IPMN as compared with non-invasive IPMN. Moreover, miR-10a-5p is highly expressed in invasive IPMN (107). MiR-99a, miR-99b, miR-100, miR-126, miR-130a, and miR-342-3p are downregulated in high-risk IPMNs. The alteration of those miRNAs may provide novel insights into miRNA-mediated formation and progression to pancreatic malignancy. Whether they could be promising potential biomarkers to distinguish between benign and invasive IPMN warrants further studies.

#### 4.1.4 MicroRNAs in MCN

The expression of miR-17-3p, miR-21, and miR-221 is significantly higher in mucinous than in non-mucinous cysts (108). MiR-224-5p expression is significantly higher while *PTEN* expression is lower in pancreatic mucinous cystadenocarcinoma cells than in normal tissues. Overexpression of miR-224-5p promotes cell proliferation and invasion. *PTEN* is the direct target gene of miR-224-5p and negatively correlates with each other (41).

### 4.2 Long non-coding RNAs

LncRNAs are RNA molecules and usually consist of more than 200 nucleotides without protein-coding potential (109).

#### 4.2.1 LncRNAs in pancreatic premalignancy

Neat1 is a large intergenic non-coding RNA (lincRNA) and is p53-regulated with a key role in suppressing transformation and tumorigenesis. Neat1 deficiency promotes the development of ADM, PanIN, and PC in *Kras^G12D^* mice (110). LncRNA *Ppp3ca* and lincRNA1611 expressions are significantly high in PC cells and tissues. *Ppp3ca* is low in normal pancreatic tissues and PanIN-I and PanIN-II tissues, while it is upregulated in PanIN-III and PC tissues (111).

LncRNA *HAND2-AS1* is a suppressor in multiple cancer (42, 112, 113). *HAND2-AS1* could regulate cytokine–cytokine receptor interaction, calcium signaling pathway, PI3K–Akt signaling pathway, and actin cytoskeleton (42). LncRNA *HAND2-AS1* and *CTD-2033D15.2* are gradually downregulated from the normal main pancreatic duct to low-grade IPMN, high-grade IPMN, and invasive IPMN, while LncRNA-TFG is gradually upregulated. LncRNAs promote tumorigenesis of IPMN and could serve as early diagnostic biomarkers and therapeutic targets in PC (114). Moreover, lncRNAs *ADARB2-AS1*, *PANDA*, *ANRIL*, *LINC00472*, *MEG3*, *PVT1*, *GLIS3-AS1*, and *UCA1h* in plasma from patients have high accuracy in discriminating between malignant and benign IPMNs (115).

#### 4.2.2 LncRNAs in PC

*N* ^6^-methyladenosine of *LINC00857* is highly upregulated and enhances its RNA stability (43). The *LINC00857* competes with endogenous RNA for sponging miR-150-5p, then upregulates its target E2F3, promotes proliferation, and inhibits apoptosis in PC cells. *DUXAP8* is upregulated whereas miR-448 is downregulated in PC tissue and cells. *DUXAP8* directly targets miR-448, which directly binds to WTAP. *DUXAP8* sponges miR-448 to modulate the malignancy of PC cells (44).

LncRNA *MALAT1* is highly expressed in PC tissues, while the large tumor suppressor 1 (*LATS1*) expression is downregulated and *YAP1* is upregulated. *MALAT1* influences proliferation, migration, and invasion in PC by regulating Hippo-YAP signaling (45). *MALAT1* also interacts with RNA binding protein HuR, dramatically enhances the regulation of TIA-1, and has further effects on inhibiting autophagy (46).

CERS6 antisense RNA 1 (*CERS6-AS1*) is highly increased in PC. *CERS6-AS1* competitively binds to miR-15a-5p as a molecular sponge in PC, increasing the expression of fibroblast growth factor receptor 1 (*FGFR1*), which also is a direct target of miR-15a-5p (47). Additionally, *CERS6-AS1* exerts as a molecular sponge for miR-217-5p. MiR-217-5p suppresses cell proliferation and metastasis by targeting YWHAG, which targets RAF1, promotes its phosphorylation, and activates RAF1-mediated ERK signaling (116). Moreover, *CERS6-AS1* sponges miR-15a-5p and miR-6838-5p to regulate HMGA1, which involves cell proliferation and migration in PC (117).

The expression of lncRNA *TP73-AS1* is upregulated and miRNA-128-3p is downregulated in PC. *TP73-AS1* promotes PC progression through binding to miR-128-3p and upregulating *GOLM1* (48). High expression of *TP73-AS1* correlates with poor overall survival. The *TP73-AS1* positively regulates BDH2 expression by sponging miR-141 (49). The *TP73-AS1* led to high MMP14 expression through miR-200a sponging, which significantly enhances migration and invasion in PC (118).

LncRNA *PART1* expression is significantly upregulated in PC and correlates with tumor size, clinical stage, and poor overall survival. *PART1* serves as a molecular sponge of miR-122. *PART1* knockdown significantly reduces cell proliferation and invasion of PC (50). LncRNA *ELFN1-AS1* is highly upregulated in PC. The knockdown of *ELFN1-AS1* significantly increases cancer cell apoptosis and growth arrest (119).

The expression level of *LINC00460* is increased in PC. Moreover, miR-320b directly targets *LINC00460*, whose knockdown leads to a downregulation in ARF1 expression. *LINC00460* modulates the miR-320b/ARF1 axis, which inhibits tumor cell proliferation, migration, and invasion (51). *LINC00460* directly targets and attenuates the tumor suppressor miR-491-5p and accelerates PC progression (120).

### 4.3 Circular RNAs

Alternative splicing of both exons and introns of the pre-mRNA can generate circRNAs. The circRNAs usually act as miRNA sponges because of their multiple miRNA response elements in the sequence. Certain circRNAs can also bind to proteins with RNA-binding sites. They can also encode for and translate into various proteins.

There are several circRNAs that are known to be important in PC development and progression. CircEYA3 is highly elevated in PC and associated with a poorer prognosis. CircEYA3 works as an endogenous miR-1294 sponge to upregulate c-Myc expression and promotes PC progression (52). CircNEIL3 is increased in PC and promotes PC progression. CircNEIL3 regulates ADAR1 expression by sponging miR-432-5p and induces the editing of glioma-associated oncogene 1 mRNA, thus affecting the cell cycle and promoting EMT in PC (121). The expression of circ_0075829 is also upregulated and regulates the LAMTOR3/p-ERK signaling pathway *via* sponging miR-1287-5p in PC. Knockdown of circ_0075829 significantly suppresses the malignancy of PC cells (53). The expression of *CircCCT3* is significantly increased in PC tissues and cell lines. Patients with high expression of *CircCCT3* have significantly poorer survival than those with low *CircCCT3* expression. Moreover, *CircCCT3* facilitates VEGFA and VEGFR2 expression as a sponge for miR-613 and promotes the malignancy of PC (54).

## 5 Alternative splicing in pancreatic premalignancy

Alternative splicing has an important role in tumorigenesis, and the splicing-derived isoforms are potentially powerful diagnostic, therapeutic, and prognostic factors. Alternative splicing produces multiple actin regulators, hMENA protein isoforms with hMENA^11a^ and hMENAΔv6 (7). hMENA is absent in normal pancreas and low-grade PanINs and very weak in PanIN-3, while high hMENA protein levels are present in PC. However, the hMENA11a isoform is only strongly expressed in 26% of PC cases. In the absence of hMENA^11a^, increased expression of hMENA and hMENAΔv6 is key to SMAD2-mediated TGF-β1 signaling and EMT. The absence of hMENA^11a^ significantly correlates with poor prognosis. The hMENA splicing and associative pathways are promising targets for the prognostic biomarkers and treatment in PC (55).

Tissue factor (TF) is a membrane glycoprotein and an enzymatic cofactor of the serine protease FVII/FVIIa and triggers the blood coagulation cascade. The murine alternatively spliced tissue factor (masTF) lacks exon 5 and has a distinct 93-amino-acid C-terminus. The intensity of masTF expression increases from early PanINs to PC phenotype, suggesting that masTF could promote PC growth (56).

The alternative splicing of FGFR-2 produces two variants: IIIb and IIIc. The expression of FGFR-2 IIIb correlates with a poor prognosis and may promote migration and invasion in PC (57). FGFR-2 IIIc is highly expressed in PC tissues and cell lines, which correlates with increased proliferation, migration, and invasion. High FGFR-2 IIIc levels in PC contribute to cancer stemness and could be a novel target for PC therapy (58).

## 6 Histone modification in pancreatic premalignancy

Epigenetic regulation can effectively change gene expression through histone post-translational modification and chromatin remodeling (122) (**Table 1**; **Figures 1**, **2**). The factors that regulate epigenetics undergo certain expression changes in ADM, leading to the silence of genes specific to acinar cells and upregulation of the genes specific to ductal cells.

### 6.1 Histone methylation

*NSD1* and *SETD2* genes encode two histone H3K36 methyltransferases and are altered in approximately 10% of PC cases. NSD1 protein expression gradually increases in PanIN, IPMN, and MCN, and plays an important role in PC. Also, NSD1 expression is significantly higher in metastatic PC than in normal pancreas and primary cancer. The expression of SETD2 significantly decreases in metastatic PC and PanIN as compared to that in the normal pancreas (59). Moreover, the expression of SETD2 is downregulated in human PC and mouse models. The decreased expression of SETD2 mainly silences *Fbxw7* through epigenetics to relieve its inhibitory effect on c-Myc (123).

The multiple endocrine neoplasia syndrome type 1 (*MEN1*) is a component of the macromolecular SET domain histone methyltransferase complex, which mediates the methylation of histone 3 on lysine 4 (60). The expression of *Men1* is significantly reduced in PC (124). The absence of *Men1* in the mouse pancreas impairs caerulein-induced pancreas regeneration and accelerates *Kras*-induced tumor formation (61). The tumor-promoting effect of *Men1* deletion is related to the overexpression of the transcription factor *Jund*. Men1 inhibits the transcription of *Jund*, and *Men1* deletion leads to *Jund* upregulation (125).

NFATc2 binds to the *p15^INK4b^* promoter site, induces the H3K9 trimethylation and heterochromatin formation, allows docking of heterochromatin protein HP1γ to the complex, and promotes PC cell growth (62). NFATc2 expression increases in PanIN lesions. Enhancer of zeste 2 homolog 2 (EZH2) is a histone methyltransferase, silences Nfatc1 epigenetically by histone methylation, and promotes acinar cell redifferentiation. However, NFATC1 is required for EZH2-mediated transcriptional activation of KRAS signaling in PC cells (126).

### 6.2 Histone acetylation

Histone acetylation is a chromatin modification and plays important roles in gene regulation, DNA replication, and DNA damage response. Pancreatic acinar cells with *Kras* mutations acquire histone H4 acetylation prior to the phenotype of premalignant lesions (127). Acetyl-CoA is upregulated in *Kras*-mutant acinar cells, and the mevalonate pathway supports ADM formation. Specific knockout of the acetyl-CoA-producing enzyme ATP-citrate lyase in the pancreas suppresses ADM and carcinogenesis. The acetyl-CoA regulates histone acetylation and facilitates cell plasticity and proliferation (63). In acute or chronic pancreatitis, inhibition of histone deacetylase (HDAC) can decrease the formation of ADM and inflammatory cell infiltration (128), suggesting that it is feasible to inhibit ADM development by modulations of epigenetics. Also, PC patients having a higher expression of HDAC1 have lower survival than those having a lower expression. High expression of HDAC1 correlates with advanced stages of and reflects the malignancy of PC (64).

### 6.3 Histone ubiquitination

The expression levels of ubiquitination significantly increase in the nucleus and cytoplasm in premalignant atypical acinar cells and cells of malignant adenocarcinoma *in situ* (129). Ring1b catalyzes histone modification H2AK119ub and silences acinar cell transcription factors, which is a key mechanism for triggering acinar cell dedifferentiation and PC development (66). Ring1b expression increases in PC (130), and knockout of acinar-specific Ring1b attenuates the formation of ADM induced by *Kras* (65).

B cell-specific Moloney murine leukemia virus insertion site 1 (BMI1) belongs to the polycomb group of proteins, including polycomb repressive complex 1, which consists of BMI1, RING1, and RING2 and acts as an E3-ubiquitin ligase. Upregulation of BMI1 in early PanINs may be a protective response to Kras-driven oxidative stress. BMI1 is highly expressed in human and murine PC (131, 132). BMI1-knockout mice have an increased expression of the Ink4a/Arf locus and a decreased β-cell mass and impaired glucose tolerance. The depletion of *Bmi1* greatly decreases the levels of H2AK119Ub at the Chk2 locus. In pancreatic carcinogenesis, the role of *Bmi1* is independent of the *Ink4a/Arf* expression, because its downregulation abrogates PanIN formation even in *Ink4a*-null animals (67). In addition, high expression of H2AK119Ub1 is significantly associated with poor prognosis and metastasis (66).

### 6.4 Chromatin remodeling

Chromatin remodeling affects gene transcription by changing the binding of transcription factors to chromosomes. BRG1 is the ATPase subunit of the SWI/SNF chromatin remodeling complex and is often inactivated in PC (68). In the presence of *Kras* oncogenic mutations, duct-specific BRG1 deletion leads to the formation of IPMN (69, 133). BRG1 binds to the *Sox9* promoter region, regulates its expression, and recruits *Pdx-1* through chromatin conformation changes (134). ARID1A, another component of the SWI/SNF complex, has also been frequently mutated in PC (70). *ARID1A* deletion changes the cancer precursor spectrum from PanIN to IPMN and accelerates the onset of invasive cancer (71).

## 7 Phosphorylation in pancreatic premalignancy

S6 ribosomal protein (S6) phosphorylation is involved in PC cell proliferation. As compared to that in normal cells, the expression of S6 phosphorylation is significantly upregulated in IPMN and is associated with glucose uptake increase in malignant cells of IPMN (9). Therefore, its expression level can be detected in pancreatic juice as a biomarker of malignancy of IPMN.

pSmad3C gradually downregulates from low-grade dysplasia and high-grade dysplasia to malignant IPMN, whereas pSmad3L upregulates. There are inverse relationships between the expression of pSmad3C and that of c-Myc, and between pSmad3C and Ki-67. There are positive relationships between the expression of pSmad3L and that of c-Myc, and between pSmad3L and Ki-67 (135).

As a member of the ezrin and moesin protein family, Ezrin acts as a cross-linker between the cell membrane and actin cytoskeleton. Ezrin is activated by threonine and tyrosine phosphorylation (72). The expression of phosphorylated ezrin (p-ezrin, tyr354) is higher significantly in IPMN than in invasive carcinoma. Moreover, p-ezrin (tyr353) is highly expressed in all grades of PanINs. The p-ezrin (tyr354) expression is associated with invasion in IPMNs, while p-ezrin (tyr353) expression plays an important role in early PanIN development (73).

## 8 Epigenetic biomarkers for early PC detection and diagnosis

**Figure 3:**
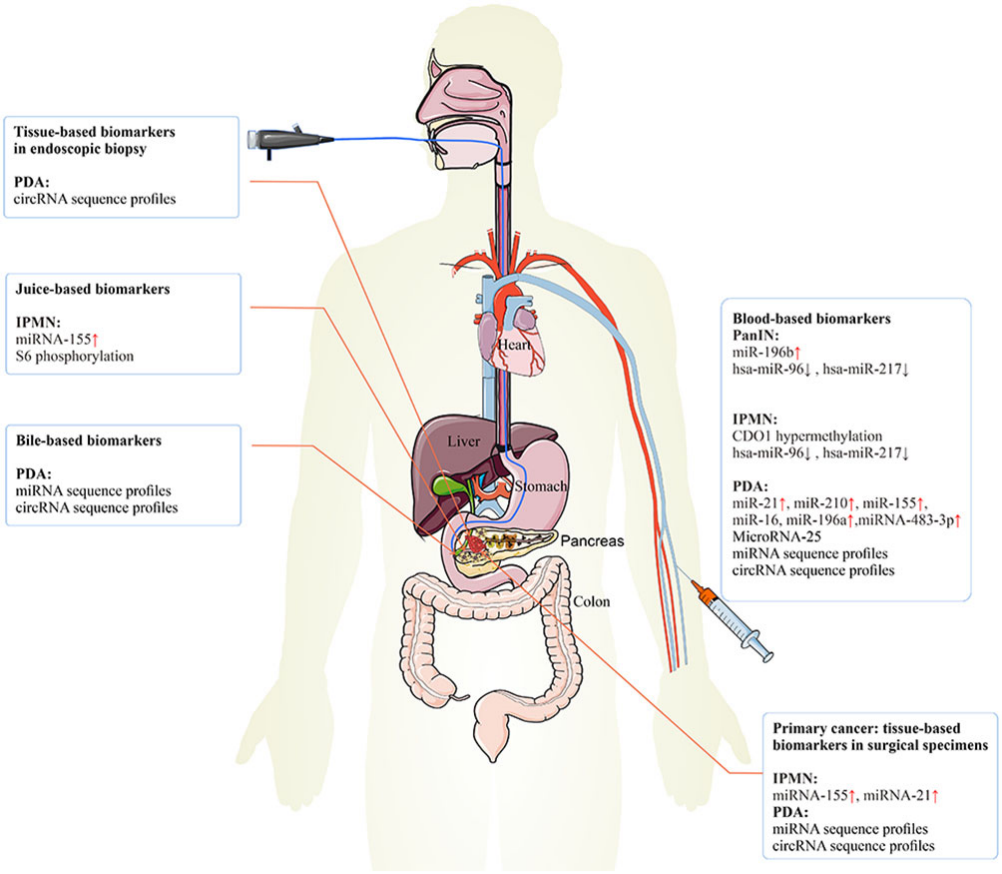
The epigenetic biomarkers for early PC detection. The epigenetic biomarkers or regulators can be exploited for clinical detection and diagnosis of early PC. Tissue-based biomarkers in endoscopic biopsy specimens may be used clinically to improve the prediction of the risk of PC.​ Tissue-based biomarkers in surgical specimens may have diagnosis value in IPMN and PC. Blood-based biomarkers may have diagnosis value in PanIN, IPMN, and PC. Juice-based biomarkers may have diagnosis value in IPMN. Bile-based biomarkers may have diagnosis value in PC. PC, pancreatic cancer; IPMN, intraductal papillary mucinous neoplasm; PanIN, pancreatic intraepithelial neoplasia.

The discovery of specific biomarkers is essential for the detection of PC premalignancy and diagnosis of PC at its early stages (136). Currently, only the serum carbohydrate antigen 19-9 (CA19-9) is a Food and Drug Administration (FDA)-approved marker for clinical treatment efficacy in PC (137). However, CA9-9 has limitations, including ineffectiveness, low sensitivity, and low specificity. Other biomarkers such as carcinoembryonic antigen (CEA) and cancer antigen 125 (CA125) are ineffective as early PC biomarkers (138). Evidently, epigenetic biomarkers have many potential advantages including stability in serum and non-invasive, economical, and convenient detection (**Table 2**; **Figures 1**, **3**).

**Table 2:** Clinical trials on epigenetic biomarkers for pancreatic cancer.

Category	Status	Biomarkers	Clinical trials identifier	Location	Year
**DNA Methylation**	Unknown	DNA hypermethylation	NCT02079363	Denmark	2014
**MiRNAs**	Recruiting	Micro-RNA profile	NCT04765410	Romania	2021
**MiRNAs**	Unknown	MicroRNA-25	NCT03432624	China	2018
**MiRNAs**	Recruiting	MiRNA profiles	NCT04406831	United States	2020
**MiRNAs**	Recruiting	MiRNA profiles	NCT02531607	United States	2015
**CircRNAs**	Recruiting	CircRNA expression profile	NCT04584996	United Kingdom	2020

### 8.1 DNA methylation biomarkers

DNA methylation changes during the development of cancer. The methylation usually occurs in the regulatory region of the gene and leads to the inactivation of the gene. Small amounts of DNA are released into the blood and can be detected in a blood sample. The DNA methylation changes can be tumor-specific and potentially usable tumor biomarkers. The alteration in hypermethylation of DNA from plasma is a diagnostic biomarker for PC (NCT02079363). Hypermethylation of cysteine dioxygenase 1 (*CDO1*) promoter is specific to IPMN and may increase with IPMN tumor progression, suggesting that *CDO1* is a potential diagnostic marker of pancreatic cystic diseases (139).

### 8.2 MiRNAs and circRNAs as biomarkers

#### 8.2.1 Plasma-associated miRNAs

Plasma miRNA profiling could be a sensitive and specific blood-based biomarker assay for PC in the clinic. MiR-155, miR-196a, miR-21, and miR-210 are implicated in PC development. Analyses of plasma levels for this panel of four miRNAs have shown certain sensitivity and specificity (140). The combination of CA19-9, miR-16, and miR-196a in plasma is more effective for distinguishing PC from non-PC (normal or chronic pancreatitis), especially in early tumor screening (141). MiRNA-483-3p and miRNA-21 from blood plasma could be PC biomarkers. MiR-483-3p expression is significantly increased in PC patients than in IPMN patients (142).

#### 8.2.2 Tissue-associated miRNAs

MiRNA-155 and miRNA-21 are also significantly overexpressed in IPMN lesions (40). Locked nucleic acid *in situ* hybridization has verified that the expression of miRNA-155 is much higher in IPMNs than in normal ducts and that the expression of miRNA-21 is much higher in IPMNs than in normal ducts. The specificity of miRNA-155 upregulation as a biomarker of IPMNs is observed in 60% of IPMN cases in pancreatic juice samples compared to 0% of healthy controls (143).

MiR-196b is overexpressed selectively in PanIN-3 and PC lesions as compared with lower-grade PanINs and normal pancreatic ducts. These samples are isolated by laser capture microdissection. The expression of miR-196b is restricted in PanIN-3 lesions and could be an early diagnostic marker (102). The expression of hsa-miR-96 and hsa-miR-217 is significantly downregulated with increasing grades of PanINs and IPMNs as compared with normal pancreatic ductal epithelium in formalin-fixed paraffin-embedded specimens. They may serve as potential early biomarkers (144).

##### 8.2.2.1 *Ongoing clinical trials* (selected data from ClinicalTrials.gov)

There are clinical trials on epigenetic biomarkers for the early detection of PC (**Table 2**). For example, *NCT04765410* analyzes miRNA profile (84 miRNAs) using qRT-PCR array in every participant to identify the correlation between microRNA expression in PC tissue and tumor aggressive behavior, treatment response, and patient survival. Samples are obtained from the participants through fine-needle aspiration during endoscopic ultrasonography. *NCT03432624* tries to improve the efficacy of PC diagnosis by combined detection of microRNA, conventional tumor markers, and imaging. *NCT04406831* determines whether the detection of certain miRNAs present in the blood of PC patients is important to the early diagnosis of the disease and whether miRNA detection in PC patients helps predict treatment response and produces prognostic information.

In the *NCT02531607* trial, the patients are assigned to control, non-carcinoma, carcinoma non-pancreatic, and pancreatic ductal adenocarcinoma. Comparative efficacy of biomarkers, brush cytology, CA19-9, and biliary and blood biomarkers including lipidomics, miRNAs, and proteomics is determined for the diagnosis of pancreatic malignancy.

In the *NCT04584996* trial, paired samples of PC tissue and associated normal pancreatic tissue will be collected after pancreatic surgery. The expression levels of circRNAs will be determined, and significantly dysregulated candidate circRNAs will be selected. Candidate circRNA expression in blood plasma samples as a clinically diagnostic biomarker in PC and superior sensitivity than serum CA19-9 will be evaluated. Moreover, the expression of candidate circRNAs in patient tissue, blood, bile, and biopsy samples will be explored as biomarkers for PC diagnosis. Finally, bioinformatic data will be reviewed to determine the abilities of miRNAs to bind candidate circRNAs.

## 9 Therapeutic implications of epigenetic regulators

Unlike genetic changes, epigenetic alterations can be easily reversed by pharmacological manipulations. Numerous studies have focused on understanding the key epigenetic regulators that regulate these epigenetic processes and developing molecule inhibitors targeting these regulators. Increasing clinical trials are conducted on epigenetic alterations as valuable targets to develop cancer therapies (**Table 3**; **Figure 1**).

**Table 3:** Clinical trials on epigenetics as therapeutic targets in pancreas cancer.

Category	Status	Drug	Combination	Clinical trials identifier	Phase	Location	Year
**DNA methylation** **Histone modification**	Recruiting	Azacitidine Romidepsin	nab-Paclitaxel Gemcitabine Durvalumab Lenalidomide capsule	NCT04257448	Phase 1 Phase 2	Germany	2020
**DNA methylation**	Active, not recruiting	Oral azacitidine	Abraxane gemcitabine	NCT01845805	Phase 2	United States	2013
**DNA methylation**	Terminated	Vidaza	–	NCT01167816	Phase 1	United States	2010
**DNA methylation**	Active, not recruiting	Azacitidine	Pembrolizumab	NCT03264404	Phase 2	United States	2017
**DNA methylation**	Completed	CC-486 (oral azacitidine)	Carboplatin ABI-007	NCT01478685	Phase 1	United States	2011
**Histone modification**	Recruiting	Tazemetostat	Durvalumab	NCT04705818	Phase 2	France	2021
**Histone modification**	Terminated	INCB054329	–	NCT02431260	Phase 1 Phase 2	United States	2015
**Histone modification**	Terminated (study terminated due to safety issues.)	INCB057643	Gemcitabine Paclitaxel Rucaparib Abiraterone Ruxolitinib Azacitidine	NCT02711137	Phase 1 Phase 2	United States	2016
**Histone modification**	Terminated	Vorinostat	5-FU	NCT00948688	Phase 1 Phase 2	United States	2009
**Histone modification**	Terminated	Vorinostat	Radiation therapy	NCT00831493	Phase 1 Phase 2	United States	2009
**Histone modification**	Not yet recruiting	ZEN003694	Entinostat	NCT05053971	Phase 1 Phase 2	United States	2021
**Histone modification**	Terminated	Bortezomib Panobinostat	–	NCT01056601	Phase 2	United States	2010
**Histone modification**	Completed	Capecitabine Vorinostat	Radiotherapy	NCT00983268	Phase 1	United States	2009
**Histone modification**	Completed	Gemcitabine hydrochloride Tacedinaline	–	NCT00004861	Phase 2	United States	2004
**Histone modification**	Active, not recruiting	Gemcitabine Vorinostat	Sorafenib Radiation	NCT02349867	Phase 1	United States	2015
**Histone modification**	Completed	Entinostat	Nivolumab	NCT03250273	Phase 2	United States	2017
**Non-coding RNA**	Completed	Atu027	Gemcitabine	NCT01808638	Phase 1 Phase 2	Germany	2013

Many clinical trials on epigenetic regulators are being carried out for treating PC according to the data from *ClinicalTrials.gov*. The DNMT inhibitors 5-azacytidine and decitabine can trap DNMT proteins during the S-phase and degrade the trapped DNMTs and hypomethylated CpG islands of tumor suppressor genes (145, 146). Azacitidine and decitabine are used for hematological cancers in clinics.

There are clinical trials on the epigenetic treatment of PC (**Table 3**). For example, *NCT04257448* aims to determine the safety and tolerability of HDAC inhibition by romidepsin and DNMT inhibition by azacitidine or both agents, in combination with gemcitabine/nab-paclitaxel in patients with advanced PC. Similarly, a phase II trial (*NCT01845805*) is ongoing to improve outcomes in patients with resected PC and high recurrence risk using oral azacitidine therapy. This trial is for patients who have resected PC and have completed adjuvant therapy or who are unable to receive adjuvant therapy due to an increased CA19-9 or advanced disease. Moreover, the phase I trial of 5-azacitidine plus gemcitabine (*NCT01167816*) is for patients having advanced PC. The primary objective is to define the maximum tolerated dose (MTD) of gemcitabine and azacitidine in patients with unresectable and previously untreated PC and to determine the effect of azacitidine therapy on DNA methylation in peripheral blood cells.

In the *NCT02847000* trial, patients with PC who have stopped responding to one or more chemotherapy drugs are asked to take part in this study on decitabine therapy that decreases DNMT1 protein level. The study hopes to find out whether decitabine will have an effect on PC. Decitabine is also being given with another drug, tetrahydrouridine (THU), to improve the exposure of the PC cells to decitabine. Phase II trials (*NCT04705818*) are conducted in parallel to assess the efficacy of durvalumab when prescribed with tazemetostat, separately, in advanced PC.

Because preclinical data in a PC mouse model demonstrates an improvement in survival with the combined use of a hypomethylating agent and immune therapy, *NCT03264404* phase II study is designed to determine the effectiveness of combining immune therapy, pembrolizumab, with a hypomethylating agent, azacitidine, for locally advanced or metastatic PC. Another phase I study (*NCT01478685*) is to use CC-486 alone or in combination with ABI-007 or carboplatin in patients with relapsed or refractory solid tumors.

The bromodomain and extra-terminal domain (BET) binds to acetylated histone lysine residues within chromatin and interacts with gene promoters/enhancers (147). After binding, BET proteins recruit transcription complexes, thereby promoting gene transcription. INCB054329 and INCB057643 are new oral BET inhibitors that are tested in phase I and II clinical trials for patients with advanced cancer (*NCT02431260* and *NCT02711137*) (148).

Vorinostat (Zolinza) is an HDAC inhibitor. In this first study (*NCT00948688*), vorinostat will be given along with radiation therapy (RT) and 5-FU to determine whether vorinostat combined with radiation and 5-FU may improve PC treatment. The phase I and II trials (*NCT00831493*) determine the MTD of vorinostat plus radiation therapy in patients with locally advanced PC. Another phase I trial (*NCT00983268*) studies the side effects and optimal dose of vorinostat when used in combination with capecitabine and radiation therapy for patients with non-metastatic PC. In the *NCT02349867* phase I study, concurrent chemoradiation uses a regimen of sorafenib and vorinostat with gemcitabine and radiation followed by chemotherapy in patients with PC to find the recommended phase II dose of the concurrent chemoradiation combination. The U.S. FDA has approved vorinostat (Merck & Co., Inc.) to treat cutaneous manifestations in patients with progressive, persistent, or recurrent cutaneous T-cell lymphoma disease or following two systemic therapies (149).

Phase II (*NCT05053971*) will determine the MTD of entinostat and BET bromodomain inhibitor ZEN-3694 (ZEN003694) in combination in patients having advanced and refractory PC, and the overall response rate of entinostat and ZEN003694 in advanced/progressive PC. Similarly, the phase II clinical trial (*NCT03250273*) of combined use of entinostat and nivolumab is for patients having metastatic or previously treated unresectable PC.

Panobinostat (LH589) produces diverse effects on endothelial cells and suppresses tumor angiogenesis. Bortezomib (Velcade) triggers cell death in PC cells. A phase II study (*NCT01056601*) of panobinostat in combination with bortezomib focuses on Patients with PC progressing on gemcitabine monotherapy or gemcitabine in combination. Moreover, a phase II trial (*NCT00004861*) compares the effectiveness of gemcitabine with or without tacedinaline (CI-994) in treating patients with advanced PC.

Finally, Atu027 is a liposomal formulation of siRNA targeting protein kinase N3 and can suppress cancer growth. *In vitro* Atu027 suppresses PKN3 function in endothelial cells and impairs their tube formation (150). Disease stabilization has been achieved in 41% of patients at end of treatment (151). In the *NCT01808638* clinical trial, combined use of Atu027 and gemcitabine appeared to be safe and well tolerated. In patients with advanced PC, twice-weekly Atu027 significantly improved outcomes (152).

## 10 Conclusion and future perspectives

Effective management of PC urgently needs early detection through the discovery of highly sensitive and specific biomarkers for pancreatic premalignancy and novel interventional strategies with precise therapeutic targets. PanIN, MCN, and IPMN are the main pancreatic premalignant lesions. PanINs are the most frequent and too small to detect clinically. Identification and validation of precursor biomarkers are fundamentally important. Epigenetics are important regulatory mechanisms in pancreatic premalignant, including cellular proliferation and apoptosis, making them appealing candidates for diagnostic, therapeutic, and prognostic biomarkers. The expression profiles of various epigenetics molecules offer an exciting and promising opportunity for biomarker development for early diagnosis of PC. In this review, we describe several epigenetic biomarkers with the potential for early PC detection and diagnosis, including methylation of *CDO1*, miR-21, miR-25, miR-155, and miR-483-3p. However, there is no sufficient evidence to support the clinical use of these candidate biomarkers because of their mediocre performance and lack of thorough validation. Nonetheless, the performance of some investigational biomarkers could be improved through combination with existing tumor biomarkers (e.g., serum CA19-9) and radiological features (137, 138).

DNA methylation is a major epigenetic program regulating gene transcription. Aberrant hypermethylation of CpG islands in the promoters/enhancers of tumor suppressor genes and hypomethylation of oncogenes are key to early tumorigenesis (153, 154). m^6^A is the most abundant reversible methylation in mammalian mRNA and plays crucial roles in tumorigenesis. Histone post-translational modification and chromatin remodeling are the most complicated types of epigenetic programs and are crucial in PC development and progression (122).

Likewise, miRNAs in pancreatic premalignancy regulate cell proliferation, growth, metastasis, invasion, and apoptosis (41, 43). The direct targeting of miRNAs provides a novel insight into the development of diagnostic biomarkers and targeted therapy of PC. Currently, only the microRNA-25 and miRNA profiles have been in clinical trials, while not a single miRNA biomarker is applied in the clinic for the detection and diagnosis of PC or premalignant lesions. Investigation into the regulation of miRNAs within pancreatic premalignant lesions using an effective and credible approach remains a major challenge. Both lncRNAs and circRNAs are emerging and promising molecules that need further systematical studies for their full potential as biomarkers in pancreatic premalignant lesions.

Preclinical studies and clinical trials have shown that epigenetic alterations are potentially reversible through the use of DNMT, EZH2, BET, and HDAC inhibitors. However, it remains to determine whether PC patients benefit from receiving epigenetic inhibitors with improved survival, and none of the available epigenetic regulators that entered clinical trials has passed phase II. Nevertheless, the potential of targeting epigenetic regulators should be systematically investigated, particularly in combination with chemotherapy, radiotherapy, immunotherapy, and targeted therapy to increase therapeutic efficacy and avoid toxicity.

With improving knowledge and understanding of the molecular basis on the initiation and progression of pancreatic premalignancy and with potential identification and validation of novel diagnostic biomarkers and therapeutic targets, it becomes possible not only for early detection and diagnosis of PC but also for screening of patients with a high risk for developing a malignant transformation and clinical management of these patients before the development of high-grade dysplasia or invasive PC.

## Author contributions

WZ, TJ, and KX reviewed the literature for the article and wrote, reviewed, and/or edited the manuscript before its submission. All authors contributed to the article and approved the submitted version.
